# Distributions, Early Diagenesis, and Spatial Characteristics of Amino Acids in Sediments of Multi-Polluted Rivers: A Case Study in the Haihe River Basin, China

**DOI:** 10.3390/ijerph13020234

**Published:** 2016-02-19

**Authors:** Yu Zhao, Baoqing Shan, Wenzhong Tang, Hong Zhang, Nan Rong, Yuekui Ding

**Affiliations:** 1 State Key Laboratory on Environmental Aquatic Chemistry, Research Center for Eco-Environmental Sciences, Chinese Academy of Sciences, Beijing 100085, China; zhaoyugreen@163.com (Y.Z.); wztang@rcees.ac.cn (W.T.); hongzhang@rcees.ac.cn (H.Z.); rongnan420@163.com (N.R.); yuekuiding@163.com (Y.D.); 2 University of Chinese Academy of Science, Beijing 100047, China

**Keywords:** Haihe River Basin, Ziya River Watershed, organic matter, amino acids

## Abstract

The Haihe River Basin, which is one of the most water-scarce and polluted river basins in China, has abnormally high nitrogen levels. In this study, total hydrolyzable amino acids (THAAs) were measured in surface sediment and sediment core samples in the Haihe River Basin to determine if amino acids were potential sources of ammonium, organic nitrogen, and organic carbon. The rivers were found to be in a state of hypoxia and contain abnormally high levels of ammonium and organic nitrogen. Additionally, NH_3_-N was the predominant form of inorganic nitrogen in the surface sediments, while organic nitrogen accounted for 92.53% of sedimentary nitrogen. THAAs-C accounted for 14.92% of the total organic carbon, while THAAs-N accounted for more than 49.59% of organic nitrogen and 45.68% of total nitrogen. The major fraction of THAAs were protein amino acids. Three sediment cores of the most heavily polluted rivers also showed high levels of THAAs. Evaluation of the degradation index (DI) of sedimentary organic matter in sediments evaluated based on the THAAs revealed that most positive DI values were found in the downstream portion of the Ziya River Watershed. Additionally, the DI of surface sediment was correlated with THAAs (*r*^2^ = 0.763, *p* < 0.001), as was the DI of sediment cores (*r*^2^ = 0.773, *p* < 0.001). Overall, amino acids in sediments were found to be an important potential source of ammonium, organic nitrogen, and organic carbon.

## 1. Introduction

Sedimentary organic matter plays an important role in sediment biogeochemical processes [1], and 30%–99% of organic matter deposited on the surface sediment is remineralized during early diagenesis [2]. Factors such as sediment physicochemical properties, water column depth, and redox conditions, especially the compositions of organic matter, influence organic matter degradation [3]. The chlorin index, which is based on degradation products of chlorophyll, has been successfully applied to estimate organic matter freshness in marine sediments [4]. Compared to chlorophyll, amino acids in sediments were more easily or directly affected by human disturbance in the polluted rivers. High total hydrolyzable amino acids concentrations have been shown to be correlated with a low chlorin index [5]. Additionally, previous studies have shown that total hydrolyzable amino acids (THAAs, amino acids in sediments after hydrolyzed) were easily degraded in sediments [6]. The proportion of precursors to decomposition products (Asp/β-Ala, Glu/γ-Aba, and (Phe + Tyr)/(β-Ala + γ-Aba)) have also been widely used to verify the decomposition and degradation status of organic matter [7,8]. Moreover, degradation indices based on amino acids were successfully developed to evaluate the degradation state of organic materials by principal component analysis [9,10]. Amino acids were studied instead of the chlorin index to evaluate the degradation status of sedimentary organic matter. Overall, these previous studies have shown that knowledge of amino acids is essential to understanding the biogeochemical process of organic matter in river ecosystems.

The Haihe River Basin in Northern China is one of the most water-scarce and polluted river basins in China, and especially high in ammonium [11,12]. In recent years, this basin has been strongly affected by anthropogenic disturbances in response to rapid urbanization and heavy industrial development. Additionally, the intensification of human activities has resulted in a large volume of nutrients entering the rivers, most of which have been deposited in the sediment [13]. Although organic matter generated by hydrophytes, plankton, bacteria, and artificial sources are the primary sources of nitrogen and organic carbon in the sediment [14], few studies have focused on the composition of organic matter in multi-polluted river sediments. In our previous study, organic nitrogen, especially THAAs-N, in the surface sediment was proved to be one of the main sources of ammonium accumulation by nitrogen mineralization [15]. Therefore, this study was designed to investigate the occurrence of different forms of amino acids in both surface sediments and sediment cores from the Haihe River Basin in order to understand its early diagenesis and spatial characteristics.

## 2. Material and Methods

### 2.1. Study Area

**Figure 1:**
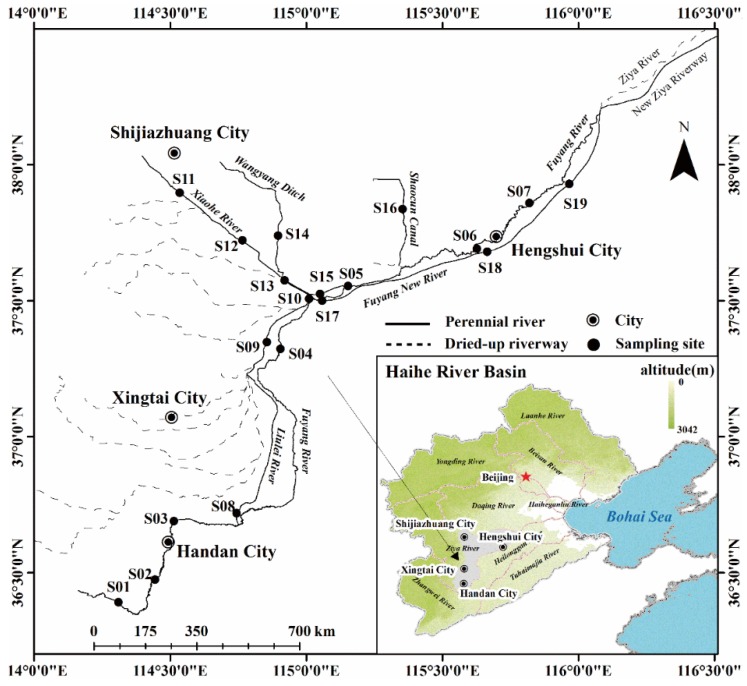
Sample sites in the plains region of the Ziya River Watershed.

The Haihe River Basin in Northern China covers 318,200 km^2^ and contains 26 cities and 1.34 billion people, making it one of the major river basins in China. The basin is characterized by a temperate semi-arid sub-humid continental monsoon climate area. The north and west portions of the Haihe River Basin consist of mountains and plateaus, while the east and southeast regions consists of plain and contain most of the cities. Rivers in the basin originate from the Yanshan and Taihang mountains. The Haihe River Basin includes nine secondary river systems and ten main rivers. The Luanhe River, Beisanhe River, Yongdinghe River, and Daqing River watersheds are located in the northern portion of the basin, while the Ziya River, Zhangwei River, Heilonggang River, and Tuhaimajia River watersheds are located in the southern portion. All rivers meet at Tianjin Province and eventually empty into the Bohai Sea (Figure 1). The largest economic region in Northern China (the Beijing-Tianjin-Hebe metropolitan area), which is known to have the worst water pollution in China, is located in the Haihe River Basin. The plain region of the Ziya River Watershed is a typical multi-polluted region.

The Ziya River Watershed, located in the south-central portion of the Haihe River Basin, is composed of two major river systems, the Fuyang and the Hutuo Rivers. After the confluence of the two rivers in Xianxian County, the Ziya River discharges into the Bohai Sea. Most parts of the Hutuohe River are located in mountain areas, while the portions in the plain generally have no flow because of construction of a reservoir and climatic conditions. The Fuyang River originates from the eastern foot of the Taihang Mountains. Below the Dongwushi Reservoir, the Fuyang River flows through the cities of Handang, Xingtai, Shijiazhuang, and Hengshui (Figure 1). Most tributaries flow into the Fuyang River at Aixingzhuang. Perennial Rivers in the plain region of the Ziya River Watershed are mainly composed of rivers in the Fuyang River system.

All perennial rivers in the plain region of the Ziya River Watershed (Fuyang River, Liulei River, Beili River, Xiaohe River, Wangyang Ditch, Shaocun Canal, and Fuyang New River) were investigated. Xiaohe River originates in Shijiazhuang and receives domestic wastewater from the surrounding cities. Wangyang Ditch receives pharmaceutical wastewater as its major water source. Shaocun Canal, which is located in the eastern of Shijiazhuang, receives leather wastewater. Samples were encoded as follows: Fuyang River upstream (S01–S04), Fuyang River downstream (S05–S07), Liulei River (S08), Beili River (S09–S10), Xiaohe River (S11–S13), Wangyang Ditch (S14–S15), Shaocun Canal (S16), and Fuyang New River (S17–S19). Surface water and surface sediment samples were collected in July 2012 (Figure 1). Additionally, three sediment cores (S13, S14, and S16) were collected from the Xiaohe River, Wangyang Ditch, and Shaocun Canal. Sample sites were mapped using Arcgis10.0.

### 2.2. Ethics Statement

No specific permissions were required for these locations, because the study area is not privately-owned or protected in any way. The field studies did not involve endangered or protected species.

### 2.3. Sample Collection and Analysis

Surface water samples were collected in 500 mL polyethylene bottles and stored at 4 °C in a cooler. Sediment samples were sampled using a Beeker-sampler, a piston corer with an inflatable valve at the bottom. After collection, the upper 10 cm were collected as the surface sediment immediately. Surface sediment samples were sealed in polyethylene bags and sediment cores were sliced at a thickness of 1.0 cm. All sediment samples were then immediately stored at −18 °C in a portable in-car refrigerator and freeze-dried under vacuum after return to the lab.

Physicochemical parameters of water samples such as temperature (T), pH, dissolved oxygen (DO) were measured by a handheld multiparameter instrument (YSI Professional Plus 556, Yellow Springs, Greene County, Ohio, OH, USA). Ammonium nitrogen (NH_3_-N), nitrite nitrogen (NO_2_^−^-N), and nitrate nitrogen (NO_3_^−^-N) were analyzed by spectrophotometric methods. Total nitrogen (TN) was determined by potassium persulfate digestion and analyzed by the spectrophotometric method. Organic nitrogen water was calculated as follows: OrgN_water_ = TN − (NH_3_-N + NO_2_^−^-N + NO_3_^−^-N). Total organic carbon (TOC) in water sample was measured using a TOC analyzer (Shimadzu TOC-VCPH, Kyoto, Japan).

Both surface sediments and sediment cores were finely ground in an agate mortar and homogenized. Inorganic nitrogen (NH_3_-N, NO_2_^−^-N and NO_3_^−^-N) was extracted at a sediment to water weight ratio of 1:10 by shaking for 1 h at room temperature with 2 mol/L KCl [16]. The supernatants of surface sediment samples were then analyzed as described above. Total Kjeldahl nitrogen (TKN) was measured using a semimicro Kjeldahl apparatus (KDY-9820). Organic nitrogen in sediment sample was calculated as follows: OrgN_sed_ = TKN−NH_3_-N. The freeze-dried sediments were acidified to remove carbonate (hydrochloric acid -treated) prior to elemental analysis, after which the total organic carbon (TOC) and TN were measured using an elemental analyzer (Model: Vario EL III; German Elementair). Three repeats were used in all experiments.

### 2.4. Amino Acids Analysis

Nineteen surface sediment samples and three sediment cores were analyzed. Amino acids in sediments were hydrolyzed and then total hydrolyzable amino acids (THAAs, amino acids in sediments after hydrolyzed) were anlayzed by the method of iTRAQ@-LC-MS/MS (isotope dilution reversed phase liquid chromatography–tandem mass spectrometry, Framingham, Massachusetts, USA) [17]. Chromatography was performed using an HPLC Ultimate 3000 system (Dionex, Sunnyvale, California, CA, USA) with an AAA C18 column (150 mm × 4.6 mm, 5 μm, AB SCIEX, Framingham, Massachusetts, MA, USA). Mass spectrometry was conducted using a SCIEX 3200QTRAP mass spectrometer (Applied Biosystems, Carlsbad, California, CA, USA). The iTRAQ^®^ kit for analysis of amino acids in sediments was provided by AB SCIEX (Framingham, Massachusetts, MA, USA). The iTRAQ^®^ kit contains all the reagents neceesary for the labeling of amino acids with the iTRAQ tag and also contains a solution of 44 amino acids labeled with the 114 iTRAQ^®^ tag (concentration of 5 μmol/L for each amino acid), to be used as internal standards. 10 mg freeze-dried sediment samples were hydrolyzed with 1 mL 6 mol/L HCl under N_2_ at 105 °C for 24 h, centrifuged, and then filtered through a 0.45 μm membrane filter. Next, 10 µL of 10% sulfosalicylic acid was added to 40 μL supernatant. The samples were subsequently vortexed for 30 s, after which the acidified supernatant was centrifuged in an Eppendorf centrifuge for 2 min at 10,000 g. Subsequently, 40 µL of borate labeling buffer containing 20 pmol/μL of norvaline were added to 10 μL of supernatant. The sample was then vortexed and centrifuged for 1 min at 10,000 g. After incubation at room temperature for 30 min, 5 μL of 1.2% hydroxylamine was added to the supernatant. Next, the sample was vortexed, centrifuged and dried under a stream of N_2_. It was reconstituted with 32 μL of iTRAQ^®^ reagent. The sample was then vortexed and centrifuged at low speed, after which it was applied to an analytical column maintained at a constant temperature of 50 °C. The mobile phase consisted of 0.1% formic acid (eluent A) and 0.001 mol/L acetonitrile containing 0.1% formic acid (eluant B). The flow rate of 0.8 mL/min and injection volume was 2 μL. Mass spectrometry was performed using the following parameters: ion spray voltage 3000 V; auxiliary gas temperature 580 °C; curtain gas, nebulizer gas, and auxiliary gas 20, 70, and 70 arbitrary units, respectively; collision gas medium; entrance potential 10 V; declustering potential 20 V; collision energy 30 V; collision cell exit potential 5 V. Quantitative determination was performed by multiple reaction-monitoring using one transition each for the analyte and the internal standard.

THAAs analysis were conducted to detect 18 species (histidine (His), arginine (Arg), lysine (Lys), aspartic acid (Asp), glutamic acid (Glu), serine (Ser), threonine (Thr), valine (Val), alanine (Ala), isoleucine (Ile), glycine (Gly), leucine (Leu), methionine (Met), tyrosine (Tyr), phenylalanine (Phe), proline (Pro), citrulline (Cit), and ornithine (Orn)). The amino acids were divided into protein amino acids, non-protein amino acids (NPAAs), and imino acids based on functional groups and chemical structure. Protein amino acids included basic (His, Arg, Lys), acidic (Asp, Glu), hydroxylic (Ser, Thr), neutral (Val, Ala, Ile, Leu, and Gly), sulfur (Met), and aromatic (Tyr, Phe) amino acids. NPAAs were composed of Orn and Cit. The imino acid was Pro.

### 2.5. Statistical Analysis

THAAs were used to evaluate the degradation of organic matter based on the degradation index (*DI*) [10,18]. *DI* was calculated using Equation (1):

(1) $$DI=\sum_{i}\left(\frac{{\mathrm{var}}_{i}-avg\;{\mathrm{var}}_{i}}{std\;{\mathrm{var}}_{i}}\right)\times fac_{i}Coe_{i}f_{i}$$

where, var is the mole percentage of individual amino acids, and *avg* var*_i_*, *std* var*_i_* and *fac_i_Coe_i_ f_i_* are the mean, the standard deviation, and the factor coefficient by principal component analysis calculated in IBM SPSS Statistics 20.

## 3. Results

### 3.1. Physicochemical Characteristics of Surface Water and Surface Sediment Samples

**Figure 2:**
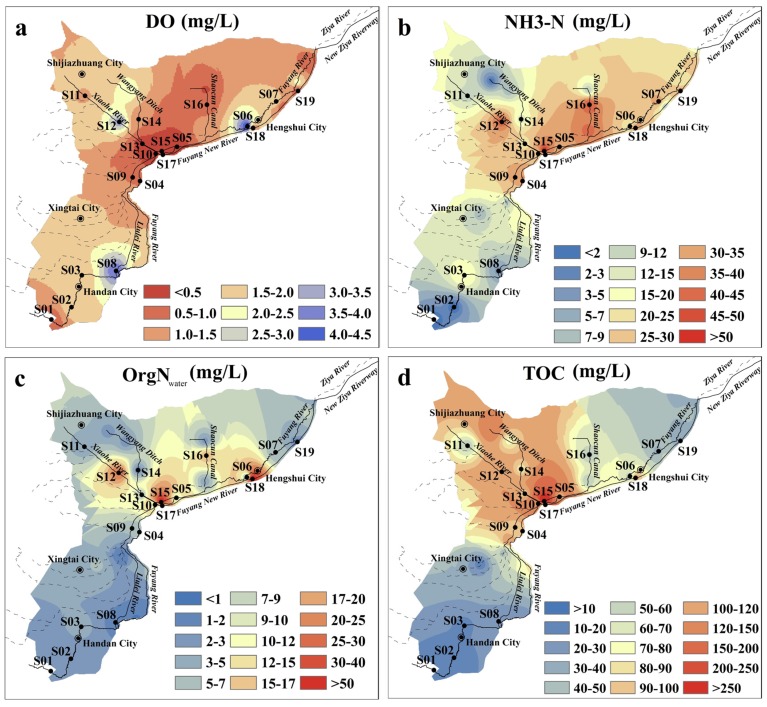
Concentrations of (**a**) DO; (**b**) NH_3_-N; (**c**) OrgN_water_; and (**d**) TOC in surface water samples of the plain region of the Ziya River Watershed.

The concentrations of DO, NH_3_-N, OrgN_water_, and TOC differed greatly in surface water collected from different regions (Figure 2). Additionally, the lowest concentration of DO and the highest concentrations of NH_3_-N, OrgN_water_ and TOC overlapped spatially. Extensive hypoxia (DO < 2.0 mg/L) occurred in the plain region of the Ziya River Watershed, especially in the Xiaohe River, Wangyang Ditch, and Shancun Canal, with the lowest DO of 0.07 mg/L being observed at S15 in Wangyang Ditch (Figure 2a). These results indicated that rivers are in an extreme state of hypoxia. The concentration of NH_3_-N in water was 21.74 ± 15.65 (mean ± SD) mg/L, and ranged from 0.16 to 69.47 mg/L (Figure 2b). NH_3_-N accounted for 67.33% of TN. A gradient change of NH_3_-N, the dominant nitrogen form, was found from upstream to downstream in the plain region of Ziya River Watershed. Most sites in the upstream portion of the Fuyang River had NH_3_-N levels lower than 9.00 mg/L, while levels as high as 26.81 mg/L were observed in downstream areas of the river. The OrgN_water_ value ranged from 0.95 to 76.50 mg/L, accounting for 23.00% of the TN (Figure 2c). The highest level of OrgN_water_ was observed in the Xiaohe River, Wangyang Ditch, and Shaocun Canal. The Xiaohe River and Wangyang Ditch also had the highest TOC at S12, S13, S14, and S15 (Figure 2d).

**Figure 3:**
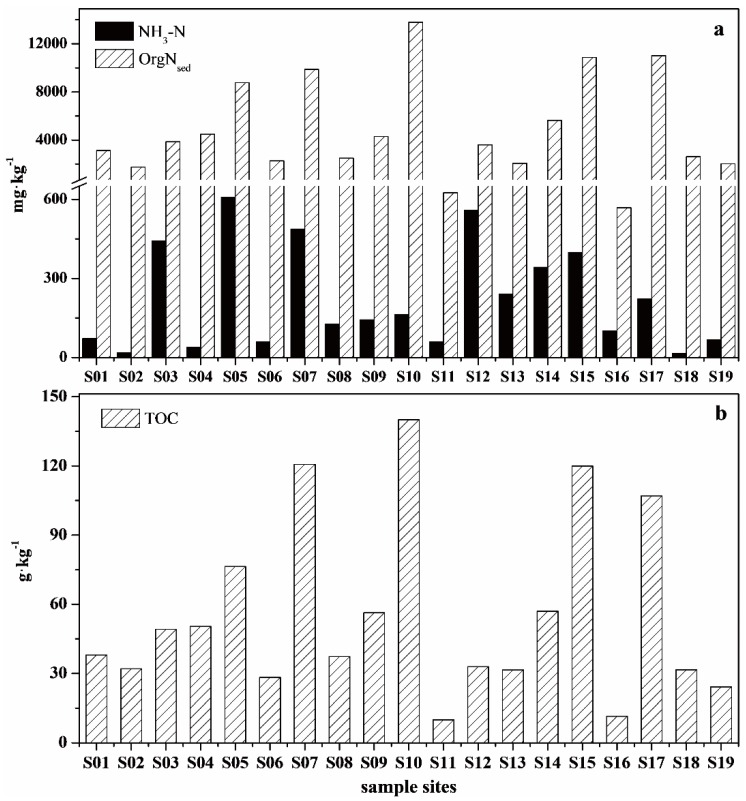
Concentrations of (**a**) NH_3_-N; OrgN_water_ and (**b**) TOC in surface sediment samples.

NH_3_-N was the predominant form of inorganic nitrogen, accounting for approximately 5.09% of nitrogen (70.33% of inorganic nitrogen) in the surface sediments of the plain region of the Ziya River Watershed (Figure 3a). The maximum content of NH_3_-N was observed in the downstream portion of the Fuyang River. The average level of OrgN_sed_ was 4932.6 mg/kg, with a range from 568.26 to 13,800.90 mg/kg, accounting for 92.53% of the sediment TN. The highest level of OrgN_sed_ was observed in the Beili River at S10, which is the point at which the Xiaohe River empties into the Beili River. The Wangyang Ditch, Beili River, downstream portion of the Fuyang River, and Fuyang New River were severely polluted by OrgN_sed_ and NH_3_-N. Sedimentary organic carbon contents gradually became worse from upstream to downstream, and the average TOC content was 55.49 g/kg (dry weight) (Figure 3b). The highest level of TOC was also observed in the Beili River. Overall, these results showed that the contents of OrgN_sed_ and TOC were similar spatially.

### 3.2. Amino Acids Concentration and Composition in Surface Sediments

**Figure 4:**
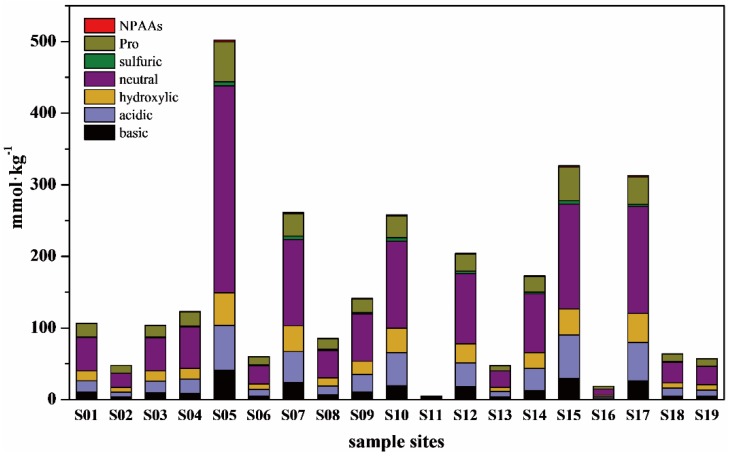
THAAs concentration in surface sediments of Ziya River Watershed.

Nineteen amino acids were detected in surface sediments from the plains region of the Ziya River Watershed (Figure 4). THAAs were divided into protein amino acids, non-protein amino acids (NPAAs), and imino acids based on functional groups and chemical structure. Protein amino acids included basic (His, Arg, Lys), acidic (Asp, Glu), hydroxylic (Ser, Thr), neutral (Val, Ala, Ile, Leu, and Gly), sulfur (Met), and aromatic (Tyr, Phe) amino acids. NPAAs were composed of Orn and Cit. The imino acid was Pro. The total concentrations of THAAs differed significantly among rivers, ranging from 4.81 to 521.69 mmol/kg, with a mean concentration of 160.25 mmol/kg. The highest concentration of THAAs appeared at S05 in the upstream portion of the Fuyang River. The lowest concentration of THAAs appeared at S11 in the Xiaohe River in Shijiazhuang. Additionally, the THAAs levels exceeded 200 mmol/kg at S07, S10, S12, S15, and S17 in the Beili River, Xiaohe River, Wangyang Ditch, Shaocun Canal, and downstream portion of the Fuyang River. Protein amino acids were the dominant THAAs. The levels of neutral amino acids, which dominated protein amino acids, ranged from 2.18 to 289.12 mmol/kg, with an average concentration of 73.45 mmol/kg. The average concentrations of acidic, hydroxylic, basic, aromatic, and sulfuric amino acids were 24.49, 18.61, 12.61, 7.67, and 1.99 mmol/kg, respectively. Among all THAAs, imino acids (Pro) were present at the highest concentration, with an average concentration of 20.59 mmol/kg. Conversely, the concentration of NPAAs was only 0.84 mmol/kg, which was much lower than the other amino acids.

The molar percentages of amino acids in these surface sediments appeared to be similar (Table 1). Ala was the second most common amino acid and the dominant protein amino acid, accounting for an average of 14.04 mol%. Gly was the third most common amino acid, accounting for 12.78 mol%, and was the second most common protein amino acid. Val, Thr, Ser, Glu, Asp, and Leu accounted for more than 5.00 mol%, while Ile, Lys, Phe, Arg, Met, His, and Tyr contributed an average of 1.00–4.00 mol%. Pro, which was the only imino acid detected, was the dominant amino acid, accounting for 14.75 mol%. The least abundant amino acids were NPAAs, with Orn and Cit contributing 0.49 and 0.10 mol%, respectively. The protein amino acids were also divided into basic, acidic, hydroxylic, neutral, sulfuric, and aromatic amino acids by function groups. Neutral amino acids accounted for 44.36 mol% of the THAAs, while acidic and hydroxylic amino acids accounted for 15.41 and 12.08 mol% and basic, aromatic, and sulfuric amino acids accounted for only 7.66, 4.29, and 1.33 mol%, respectively. There were also small differences in the amounts of various amino acids between these river reaches.

Despite close similarities to watershed scale, amino acids between river reaches and stations still showed differences among THAAs (Table 1). As the dominant protein amino acid, Ala was present at the highest levels at S11 (22.22 mol%) and S16 (20.38 mol%). The lowest level of Ala was observed in the downstream portion of Fuyang River at S05. Gly, which was the second most common protein amino acid, accounted for 12.00 mol% at most stations, except S05. Unlike the other stations, S05 also had the highest level of Leu. Met was not detected at S02, S11, S13, S16, and S19, which were located in the downstream portion of the Ziya River watershed. Pro, which was the dominant amino acid, was found at the highest levels at S02, S06, and S16. The nonprotein amino acids appeared in the lowest levels, with Cit (0.10 mol%) being less abundant than Orn (0.49 mol%). Cit was only detected at eight stations and Orn was not detected at S11 and S16.

### 3.3. Amino Acids in Sediment Cores

**Figure 5:**
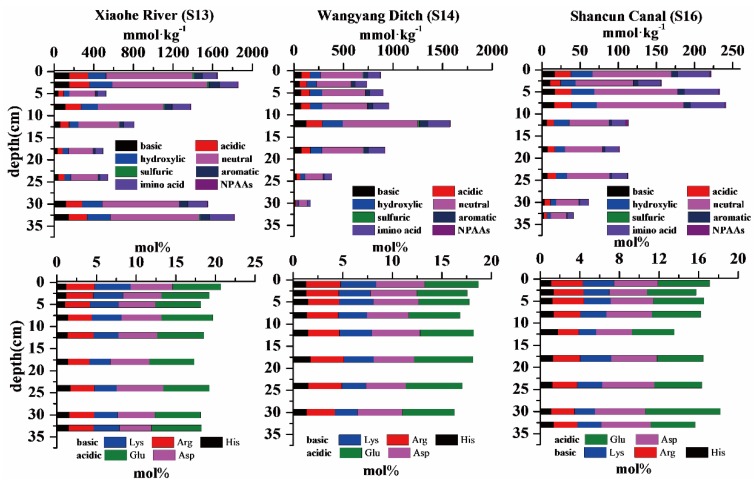

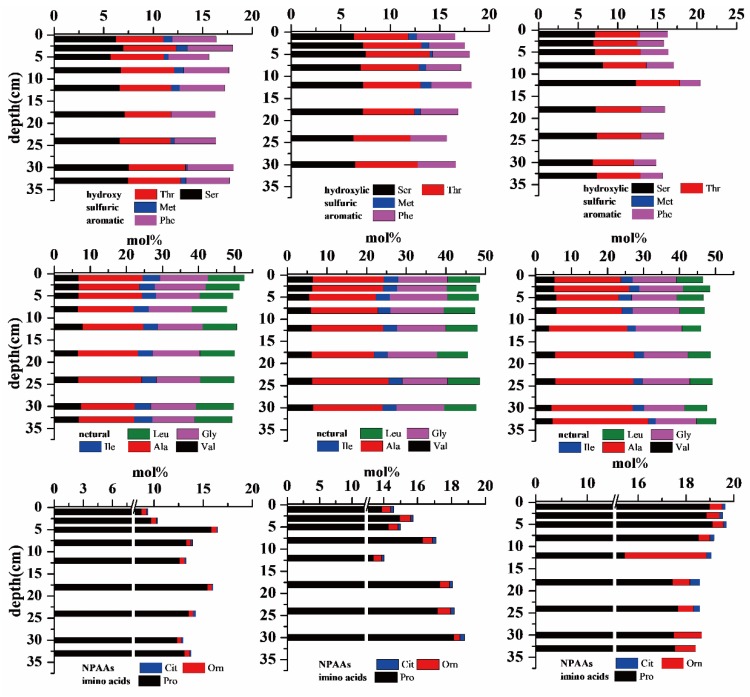
Concentration of THAAs and mole percent of amino acids in sediment cores.

The concentrations of THAAs and mole percentages of amino acids in sediment cores are presented in Figure 5. Considerable differences were observed spatially and among rivers. The average THAAs concentration was lowest at S16 (Shaocun Canal) and highest at S13 (Xiaohe River). The THAAs concentrations at S13 and S14 reached 1182.62 and 815.50 mmol/kg. When compared with S13 and S14, the THAAs were only 142.75 mmol/kg at S16, which was far lower than that of the stations in Xiaohe River and Wangyang Ditch. Vertically, three sediment cores showed different trends. The THAAs concentrations in the Xiaohe River (S13) decreased gradually from 0 to 25 cm and then increased to 1823.90 mmol/kg at 33 cm. The THAAs concentrations in the Wangyang Ditch (S14) increased slowly from 0 to 11 cm and then decreased gradually from 11 to 30 cm. The THAAs concentration in the Shaocun Canal decreased gradually from 0 to 33 cm. The highest value of THAAs appeared in the 0–3 cm sediment layer of the Xiaohe River. According to the functional group classification, the THAAs concentration of the Xiaohe River was clearly dominated by neutral amino acids (594.52 ± 296.98 mmol/kg), followed by hydroxylic amino acids (143.72 ± 74.54 mmol/kg), and imino acid (141.77 ± 60.38 mmol/kg). The results observed for the Wangyang Ditch and Shaocun Canal were similar to that observed for Xiaohe River.

All sediment cores were analyzed for THAAs. Basic amino acids, including His, Arg, and Lys, showed little changes and differences with depth among the studied cores. Similar trends were observed for acidic amino acids, including Asp and Glu. Ser, a hydroxylic amino acid, showed fluctuation with depth. Thr, another hydroxylic amino acid, showed little change at S16 and gradually increased at S13 and S14. Neutral amino acids included Val, Ala, Ile, Gly, and Leu. Ala dominated the THAAs pool in all sediment cores, followed by Pro, Gly, Ser, Leu, Thr, and Glu. The mean mole percent of Ala at S13, S14, and S16 accounted for 16.57, 17.52, and 21.05 mol%, respectively. Ala decreased gradually at S13 (Xiaohe River), fluctuated at S14 (Wangyang Ditch) and increased gradually at S16 (Shaocun Canal). Gly, which was the third most prevalent amino acid, decreased gradually in all sediment cores. Gly accounted for 12.53 mol% of the THAAs. Leu decreased gradually at S13, and all other sediment cores. Pro, the second most common amino acid, decreased at S16 and increased gradually at S13 and S14. The non-protein amino acids (Orn and Cit) did not show clear trends in depth.

## 4. Discussion

### 4.1. Contribution of THAAs to Organic Matter in Surface Sediments

The relationships between various nitrogen forms, THAAs and TOC were analyzed by Pearson’s correlation analysis (Table 2). Organic nitrogen, especially amino acids, were an important source of nitrogen and organic carbon. TN was correlated with OrgN_sed_, TOC, THAAs-C and THAAs-N (*p* < 0.01), indicating that organic nitrogen was the major form of nitrogen and that THAAs were a primary contributor. TOC was correlated with TN, OrgN_sed_, THAAs-C, and THAAs-N (*p* < 0.01), which indicated that organic carbon and organic nitrogen, had a similar source. Additionally, correlations between NH_3_-N and TN, NH_3_-N and OrgN_sed_, NH_3_-N and THAAs were significant (*p* < 0.05).

OrgN_sed_ accounted for 92.53% of the sedimentary nitrogen. The main source of ammonia in sediments was organic nitrogen formed by nitrogen mineralization. The efficiencies of particulate organic nitrogen mineralization under aerobic and anaerobic conditions were similar, but the efficiency of dissolved organic nitrogen mineralization under aerobic conditions was much greater than that observed under anaerobic conditions [19]. Moreover, the decomposition of organic nitrogen by the way of nitrogen mineralization caused a hypoxic zone at the bottom of the river [20]. Ammonium accumulation occurred in the oxygen minimum zones through mineralization of organic matter [21]. Amino acids, which were mainly in the form of protein amino acids, are the major components of sedimentary organic nitrogen, and the rates of organic nitrogen mineralization appear to be amino acid nitrogen > amino sugar nitrogen > heterocyclic nitrogen [22,23].

Amino acids, the most easily mineralized organic matter, were positively correlated with the nitrogen mineralization rate. Evaluation of the surface sediments of the Ziya River Watershed revealed that THAAs-C accounted for 14.92% of the TOC, and that the major fraction of THAAs-C were protein amino acids. THAAs-C accounted for 2.38% to 40.94% of the TOC, and comprised an average of 12.00% of the TOC in most surface sediments. When compared with TOC, OrgN_sed_, especially THAAs-N, was the major nitrogen form in the surface sediments. Moreover, OrgN_sed_ was present at higher levels than in previous studies (3.0%–17.3%) [14,24,25]. THAAs-N accounted for more than 49.59% of the OrgN_sed_ and 45.68% of the TN. The major fraction of THAAs-N was also protein amino acids. Among THAAs in the Ziya River Watershed, Pro was present in the greatest abundance, followed by Val, Gly, Leu, Asp, and Glu (Figure 4). Different from previous studies, Gly was the dominant amino acid in the other studied regions [10,26]. Proline, which is essential for primary metabolism, is synthesized from glutamate in plants [27]. Proline accumulation has been reported to be related to salinity, drought, heavy metals, anaerobiosis, temperature, nutrient deficiency, and atmospheric pollution in plants [28,29,30,31]. Additionally, Proline accumulation has been shown to play an adaptive roles in plant stress tolerance, and to help buffer pH and balance cell redox status [32]. The high percentage of Pro was consistent with the region seriously polluted by heavy metals and nutrients [12,33], and was related to the degradation of riparian or aquatic plant residues. The second most common amino acid in the Ziya River Watershed was Val, which is an essential amino acid along with Leu. Valine accumulation has been shown to occur during fermentation when the dissolved oxygen was 5% [34], which is similar to the studied region. Glycine was also a major amino acid in the Ziya River Watershed. The enrichment of Gly has been shown to be related to food and natural faunal tissue, and has been found in fecal matter and sediments because of its low nutritional value [8,35,36]. Leucine is easily assimilated by bacteria, but fungi and protozoa may also contribute significantly to the uptake of Leu in sediments [37].

### 4.2. Degradation State in Surface Sediments and Sediment Cores

**Figure 6:**
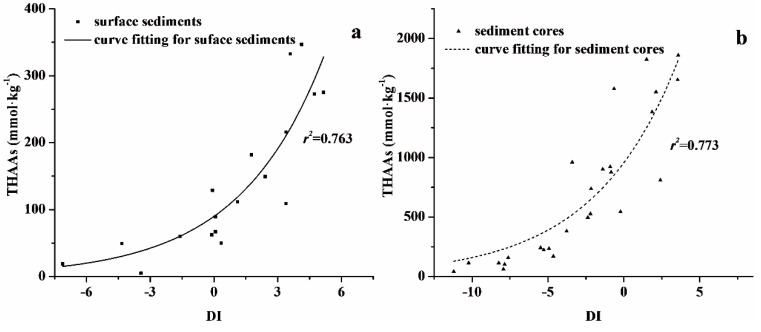
Relationship between THAAs and DI in (**a**) surface sediments and (**b**) sediment cores of the Ziya River Watershed.

The degradation index (DI) based on the composition of amino acids was used to determine the degradation status of sedimentary organic matter. A positive DI indicates fresh organic matter and a negative DI indicates degraded organic matter [38]. Most of the positive DI values were observed in the downstream portion of the Ziya River Watershed (Figure 6). The DI for surface sediments ranged from −13.52 to 5.16, while that for sediment cores ranged from −11.21 to 3.59. The lowest DI of surface sediments was observed in S05, where the Xiaohe, Beili, and Fuyang Rivers converge. The highest DI of surface sediments appeared in S07, which was located in the downstream portion of the Fuyang River. The DI values of surface sediments at S02, S04, S05, S06, S11, and S16 were negative, indicating that organic materials were degraded. The DI of surface sediments at the other stations were positive, suggesting organic materials were fresh and had a potential risk of degradation. The DI values of sediment cores in Wangyang Ditch and Shaocun Canal were negative, while the DI of sediment cores in Xiaohe River were positive. These findings implied that organic materials in the sediment cores of Wangyang Ditch and Shancun Canal were degraded and released inorganic nitrogen or other components such as NH_3_-N and TOC into the overlying water via mineralization. Organic materials in the sediment cores of Xiaohe River were fresh and had a potential risk of degradation via mineralization and release into overlying water by diffusion.

In the Ziya River Watershed, the DI and THAAs of surface sediments and sediment cores were found to have a positive relationship with an exponential fit. As shown in Figure 6a, DI was correlated with THAAs (*r*^2^ = 0.763, *p* < 0.001). The content of THAAs increased with DI, indicating that changes between components of amino acids in surface sediments could reflect the degree of sedimentary organic matter degradation. The DI of sediment cores was also correlated with THAAs (*r*^2^ = 0.773, *p* < 0.001), which indicated that DI reflected the degradation state of organic matter in sediment cores (Figure 6b).

## 5. Conclusions

Water in rivers of the plain region of the Ziya River Watershed was polluted by abnormally high levels of ammonium, organic nitrogen, and organic carbon. In this study, amino acids of sediments in the plain region of Ziya River Watershed were observed and found to be an important potential source of ammonium, organic nitrogen, and organic carbon. Organic nitrogen was the dominant nitrogen form in surface sediments, while NH_3_-N in the surface sediment was the predominant form of inorganic nitrogen, accounting for approximately 5.09% of nitrogen (70.33% of inorganic nitrogen). THAAs-N, which is an easily degraded form of organic nitrogen, was significantly correlated with TOC, OrgN_sed_, and NH_3_-N. Evaluation of the degradation states of organic matter in sediments based on the degradation index revealed that most positive DI values appeared in downstream areas, which was consistent with the levels of organic nitrogen, organic carbon, and THAAs in surface sediments of the Ziya River Watershed.

## Figures and Tables

**Table 1:** Mole percent composition of amino acids in surface sediments of the Ziya River Watershed.

Sample	mol%																	
sites	His	Arg	Lys	Asp	Glu	Ser	Thr	Val	Ala	Ile	Gly	Leu	Met	Tyr	Phe	Pro	Orn	Cit
S01	0.98	3.50	5.01	6.95	7.09	5.33	7.12	4.94	13.56	3.37	13.25	6.70	0.81	1.45	2.96	16.31	0.46	0.21
S02	0.92	2.32	3.58	7.65	6.83	7.08	5.80	4.99	13.66	3.29	12.52	5.64	nd	nd	2.74	22.56	0.42	nd
S03	1.23	3.26	4.71	7.53	7.01	6.76	6.44	5.56	13.81	3.19	12.98	7.01	1.18	0.92	3.22	14.73	0.46	nd
S04	1.27	2.70	2.58	8.44	7.32	5.42	6.01	5.17	13.66	4.20	13.91	8.08	1.09	0.96	3.54	15.16	0.48	nd
S05	1.68	2.84	3.30	5.74	6.33	4.38	4.35	3.97	9.43	3.10	8.95	29.96	1.06	1.42	2.35	10.66	0.41	0.07
S06	0.77	2.73	4.35	7.77	7.54	4.98	6.80	5.76	12.82	3.74	11.93	7.36	1.16	0.67	3.11	18.02	0.50	nd
S07	1.81	2.97	3.86	8.41	7.29	7.10	6.06	4.81	12.95	4.45	13.30	8.28	1.71	1.30	3.71	11.39	0.52	0.06
S08	0.94	2.41	4.16	6.92	6.76	6.62	6.38	5.54	14.34	3.64	12.70	6.52	1.88	0.70	3.37	16.51	0.60	nd
S09	1.09	2.77	3.39	8.90	7.26	5.29	7.26	5.93	13.73	4.57	11.77	8.20	1.43	1.50	3.80	12.60	0.53	nd
S10	1.67	2.83	2.66	9.28	7.67	6.42	6.10	5.73	12.65	4.39	13.18	8.51	1.89	1.64	3.68	11.17	0.44	0.09
S11	1.26	2.68	3.95	10.16	7.49	7.42	4.34	3.44	22.22	nd	14.69	5.05	nd	nd	2.23	15.06	nd	nd
S12	1.84	2.86	3.66	8.36	6.91	5.87	6.64	5.52	14.45	4.47	12.82	8.28	1.58	1.75	3.49	11.10	0.41	nd
S13	1.17	2.87	3.05	7.93	7.21	5.60	7.04	5.66	14.64	4.20	13.55	8.11	nd	0.70	3.41	14.54	0.30	nd
S14	1.35	2.42	3.29	9.74	7.02	6.50	5.93	5.72	13.63	4.43	13.31	8.06	1.18	1.28	3.46	12.13	0.49	0.06
S15	2.12	3.18	3.28	9.04	8.37	5.91	4.58	5.15	12.25	4.48	12.21	8.18	1.38	2.48	3.32	13.52	0.45	0.09
S16	0.94	2.56	2.60	8.41	6.25	6.26	5.09	5.22	20.38	2.48	11.82	5.93	nd	0.22	2.56	19.28	nd	nd
S17	1.82	3.11	2.89	9.33	6.81	6.70	5.47	5.81	12.78	4.78	12.88	8.62	0.95	2.14	3.81	11.52	0.54	0.04
S18	0.93	2.57	3.27	9.23	7.66	5.57	5.71	5.00	12.96	4.34	12.77	7.88	1.37	0.49	3.49	15.88	0.70	0.17
S19	1.20	3.06	3.27	7.52	6.66	6.17	6.97	4.70	12.79	4.00	14.23	7.15	nd	0.76	2.87	18.12	0.54	nd

nd: Not detected.

**Table 2:** Relationships among various nitrogen forms, THAAs-N, THAAs-C, and TOC.

Chemical Index	NH_3_-N	TN	OrgN_sed_	TOC	THAAs-C	THAAs-N
NH_3_-N	1	0.529 *	0.475 *	0.437	0.716 *	0.679 *
TN		1	0.998 **	0.978 **	0.839 **	0.872 **
OrgN_sed_			1	0.982 **	0.817 **	0.854 **
TOC				1	0.744 **	0.791 **
THAAs-C					1	0.994 **
THAAs-N						1

* *p* < 0.05, ** *p* < 0.01.
